# Slow Inactivation of Sodium Channels Contributes to Short-Term Adaptation in Vomeronasal Sensory Neurons

**DOI:** 10.1523/ENEURO.0471-21.2022

**Published:** 2022-05-13

**Authors:** Nicole Sarno, Andres Hernandez-Clavijo, Anna Boccaccio, Anna Menini, Simone Pifferi

**Affiliations:** 1 Neurobiology Group, SISSA Scuola Internazionale Superiore di Studi Avanzati, 34136 Trieste, Italy; 2 Institute of Biophysics, National Research Council, 16149 Genova, Italy; 3 Department of Experimental and Clinical Medicine, Università Politecnica delle Marche, 60126 Ancona, Italy

**Keywords:** adapation, sodium channels, vomeronasal

## Abstract

Adaptation plays an important role in sensory systems as it dynamically modifies sensitivity to allow the detection of stimulus changes. The vomeronasal system controls many social behaviors in most mammals by detecting pheromones released by conspecifics. Stimuli activate a transduction cascade in vomeronasal neurons that leads to spiking activity. Whether and how these neurons adapt to stimuli is still debated and largely unknown. Here, we measured short-term adaptation performing current-clamp whole-cell recordings by using diluted urine as a stimulus, as it contains many pheromones. We measured spike frequency adaptation in response to repeated identical stimuli of 2–10 s duration that was dependent on the time interval between stimuli. Responses to paired current steps, bypassing the signal transduction cascade, also showed spike frequency adaptation. We found that voltage-gated Na^+^ channels in VSNs undergo slow inactivation processes. Furthermore, recovery from slow inactivation of voltage-gated Na^+^ channels occurs in several seconds, a time scale similar to that measured during paired-pulse adaptation protocols, suggesting that it partially contributes to short-term spike frequency adaptation. We conclude that vomeronasal neurons do exhibit a time-dependent short-term spike frequency adaptation to repeated natural stimuli and that slow inactivation of Na^+^ channels contributes to this form of adaptation. These findings not only increase our knowledge about adaptation in the vomeronasal system, but also raise the question of whether slow inactivation of Na^+^ channels may play a role in other sensory systems.

## Significance Statement

Most mammals use neurons of the vomeronasal organ for the detection of pheromones, but whether these neurons adapt to stimuli is still subject of debate. Here, we measured spike frequency adaptation to repeated identical pulses of urine. Surprisingly, we also found that adaptation also occurred when spiking activity was induced by current steps, bypassing the transduction cascade. Analysis of action potential machinery indicates that slow inactivation of Na^+^ channels contributes to short-term spike frequency adaptation to repeated stimuli.

## Introduction

In sensory systems, the process of adaptation plays an important physiological role as it reduces the responsiveness to a stimulus, allowing the detection of additional variations in stimulus intensity. It often originates at the peripheral region of sensory systems, at the level of receptor cells, and further develops in the CNS (Torre et al., 1995; Wark et al., 2007; De Palo et al., 2012; Webster, 2012).

Chemosensory systems allow the organism to detect chemicals from the external world, and most mammals use at least two different olfactory systems: the main and the vomeronasal olfactory system. Pheromones are proteins or small molecules released from animals that affect the physiology and/or behavior of members of the same species (Wysocki et al., 1982; Tirindelli et al., 2009; Touhara and Vosshall, 2009; Brennan, 2010; Tirindelli, 2021). The vomeronasal system plays a very important role in the detection of pheromones and the regulation of several behaviors, including mating, care of pups, and aggressive and territorial behaviors (Wysocki et al., 1982; Stowers et al., 2002; Tirindelli et al., 2009; Touhara and Vosshall, 2009; Kaur et al., 2014; Mohrhardt et al., 2018; Tirindelli, 2021).

Pheromones and other natural ligands enter the vomeronasal organ (VNO) and contact receptors in vomeronasal sensory neurons (VSNs), bipolar neurons that have a dendrite ending in microvilli inside the lumen of the VNO and an axon projecting to the accessory olfactory bulb (AOB). Each VSN expresses one or a few types of G-protein-coupled receptors V1Rs, V2Rs, FPRs, and axons of VSNs expressing the same receptor project to various glomeruli in the AOB (Dulac and Axel, 1995; Herrada and Dulac, 1997; Matsunami and Buck, 1997; Liberles et al., 2009; Tirindelli et al., 2009; Francia et al., 2014; Tirindelli, 2021). Ligand binding activates a phospholipase C signaling cascade that leads to activation of transient receptor potential canonical 2 (TRPC2) channels allowing the influx of Na^+^ and Ca^2+^ and the subsequent activation of the Ca^2+^-activated Cl^–^ channels TMEM16A and TMEM16B (Liman and Buck, 1994; Lucas et al., 2003; Dibattista et al., 2012; Amjad et al., 2015; Leinders-Zufall et al., 2018; Hernandez-Clavijo et al., 2021). Ligand binding also causes Ca^2+^ release from intracellular stores that may also directly activate Ca^2+^-activated Cl^–^ channels (Kim et al., 2011). Currents induced by the transduction cascades in response to chemical stimuli generate membrane depolarization and action potentials that are sent to the AOB (Mohrhardt et al., 2018).

Although the process of adaptation in the main olfactory system has been extensively investigated both at the peripheral and central level, very little is known about adaptation in the vomeronasal system (Abbas and Vinberg, 2021; Brandão et al., 2021; Martelli and Storace, 2021). Indeed, adaptation in the vomeronasal organ has been the subject of some debate (Holy et al., 2000; Nodari et al., 2008; Spehr et al., 2009), but recent work showed additional evidence of adaptive responses of VSNs to natural stimuli (Silvotti et al., 2018; Wong et al., 2018). Repeated stimulations showed that VSNs present both short-term adaptation, in a time range of tens of seconds, and long-term adaptation, in a time range of tens of minutes (Wong et al., 2018).

In this study, we used current-clamp whole-cell recordings to investigate short-term adaptation by measuring the effect of a first stimulus on the spiking response elicited by a second identical stimulus repeated at different time intervals. We compared both responses to paired stimuli to diluted urine and to current steps, to bypass the transduction cascade. Furthermore, we analyzed the adaptation properties of voltage-gated channels independent of stimuli. We found that slow inactivation of Na^+^ channels in mouse VSNs contributes to spike frequency adaptation to repeated stimuli.

## Materials and Methods

### Animals and ethical approval

Mice were handled according to the guidelines of the Italian Animal Welfare Act (Decreto legislativo 26/2014) and European Union guidelines on animal research (2010/63) under a protocol approved by the Italian Ministry of Health. Mice had free access to water and food. Every effort was made to reduce the number of animals used. C57BL/6 mice (age range, 2–3 months) were anesthetized with CO_2_ and decapitated before removal of the VNO. Mice were transferred to a cage (height/width/length, 8 × 10 × 12 cm), and 100% CO_2_ was slowly injected into the cage until the animal stopped breathing and no longer displayed pedal reflex (near 3 min; gas flow rate, ∼20% of chamber volume/min).

### Acute slices of mouse VNO

Acute slices of mouse VNO were prepared as previously described (Shimazaki et al., 2006; Dibattista et al., 2012; Wong et al., 2018; Hernandez-Clavijo et al., 2021). The VNO was removed and transferred to ice-cold artificial CSF (ACSF). The capsule and all cartilaginous tissues were removed with the help of the tip of fine forceps obtaining the two clean halves of the VNO. Each half was embedded in 3% low-grade agar (catalog #A7002, Sigma-Aldrich) prepared in ACSF, once the agar had cooled to 38°C. Upon solidification, the agar block was fixed in a glass Petri dish filled by ice-cold oxygenated ACSF solution and sliced with a vibratome (Vibratome 1000 Plus Sectioning System, Vibratome Company) at 200 μm. Slices were then left to recover for >30 min before electrophysiological experiments were initiated.

### Electrophysiological recordings and solutions

Slices were viewed with an upright microscope (Zeiss Axioscope) with a water-immersion 40× objective (Olympus). The slice preparation maintained the VNO cross-sectional structure, and VSNs could be distinguished by their morphology. Patch pipettes, pulled from borosilicate capillaries (WPI) with a PC-10 puller (Narishige), had a resistance of 3–6 MΩ. Electrophysiological recordings were made using an Axopatch 200B amplifier controlled by Clampex 10 via a Digidata 1440A digitizer (Molecular Devices). Experiments were performed at room temperature (20–25°C). The recording chamber was continuously perfused by gravity flow with oxygenated (95% O_2_ and 5% CO_2_) ACSF containing the following (in mm): 120 NaCl, 20 NaHCO_3_, 5 KCl, 2 CaCl_2_, 1 MgSO_4_, 10 HEPES, and 10 glucose, pH 7.4. The slice was anchored to the base of the recording chamber using a home-made U-shaped silver wire, holding down the agar support without touching the slice itself.

For current-clamp recordings, the intracellular solution filling the patch pipette contained the following (in mm): 80 K-gluconate, 60 KCl, 2 Mg-ATP, 10 HEPES, and 1 EGTA, adjusted to pH 7.2 with KOH. Paired-pulse experiments were conducted at a 0 pA holding current with stimulus of variable duration (from 2 to 10 s) consisting in either diluted urine application or in 5 pA current steps. Interpulse intervals (IPIs) varied from 2 to 60 s. The time between independent sweeps was at least 2 min to avoid cumulative adaptation (Wong et al., 2018). Data were low-pass filtered at 2 kHz and sampled at 10 kHz.

**Figure 1.**
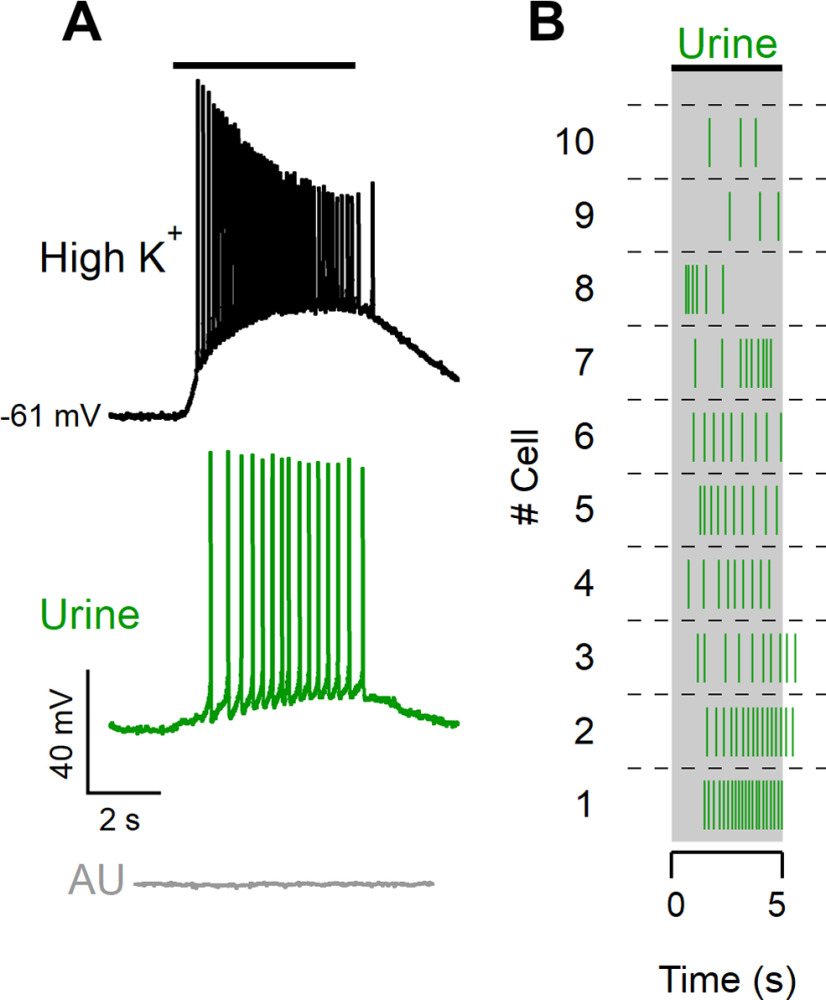
Diluted urine elicits spike activity in VSNs. ***A***, Whole-cell current-clamp recordings from a VSN stimulated for 5 s with a high-K^+^ solution (25 mm KCl; black trace), diluted urine (1:50; green trace), or diluted artificial urine (1:50; gray trace). The black bar indicates the arrival of the solutions to the neuron based on the response to the high-K^+^ solution. ***B***, Raster plot of urine responses of 10 different neurons stimulated for 5 s.

Urine was collected from both sexes of BALB/c mice, filtered with a 0.2 μm filter, and frozen at −80°C for no more than 2 months. Before use, male and female urine samples were mixed in a 1:1 ratio, and the mixture was diluted to 1:50 in ACSF, pH 7.4. As mouse urine contains urea and K^+^, which could potentially cause neurons to fire by direct membrane depolarization, we used as a negative control artificial urine diluted to 1:50 in ACSF. Artificial urine contained the following (in mm): 100 NaCl, 40 KCl, 20 NH_4_OH, 4 CaCl_2_, 2.5 MgCl_2_, 15 NaH_2_PO_4_, 20 NaHSO_4_, and 333 urea, at pH 7.4 adjusted with NaOH (Holy et al., 2000; Wong et al., 2018; Hernandez-Clavijo et al., 2021). To test cell viability, we used a high-K^+^ solution (25 mm KCl) by replacing equimolar amounts of NaCl with KCl in ACSF. Urine, artificial urine, and high-K^+^ solutions were delivered through an 8-into-1 multibarrel perfusion pencil connected to a ValveLink8.2 pinch valve perfusion system (AutoMate Scientific). For each experiment, the response to high-K^+^ stimulation (Fig. 1) was used to evaluate the arrival of solution to the cell. We included in the dataset only neurons that fired in response to high-potassium solution and did not respond to artificial urine.

For voltage-clamp recordings, the intracellular solution filling the patch pipette contained the following (in mm): 135 CsCl, 5 NaCl, 10 HEPES, and 10 EGTA, adjusted to pH 7.2 with NaOH. CdCl_2_ (100 μm) was added to extracellular ACSF in all recordings, unless otherwise stated, to block Ca^2+^ channels. TTX (2 μm; LATOXAN) was used to block Na^+^ channels. Leakage and capacitive currents were subtracted. All compounds and chemicals were obtained from Sigma-Aldrich, unless otherwise stated.

### Data analysis

IgorPro 6.7/8 software (WaveMetrics) was used for data analysis and figure preparation. Individual spikes were identified by a custom-written detection procedure and confirmed by shape inspection. Data are presented as the mean ± SD and the number of cells (*n*). The voltage-gated inactivation curves were fitted to the following Boltzmann equation: *I*/*I*_max_ = *A* + (1 – *A*)/(1+ exp((*V* – *V*_half_)/*k*), where *I* is the peak sodium current, *I*_max_ the maximal peak sodium current, *V* is the membrane potential, *V*_half_ is the membrane potential at which *I* is half of *I*_max_, *k* is the slope constant, and *A* is the asymptotic value. Statistical significance was determined using the following tests. The Shapiro–Wilk test was used to verify data normality. For normally distributed data, repeated measures ANOVA was used, followed by a paired *t* test with Bonferroni correction; while for data not normally distributed, statistical significance was determined using a Friedman’s test followed by a Demsar’s test. The *p* values associated with the Demsar’s test were calculated using the algorithm in R developed by Eisinga et al. (2017). A paired Dunnett’s test was used after ANOVA (see Fig. 8, data). A *p* value of <0.05 was considered statistically significant.

## Results

We performed current-clamp whole-cell recordings from the soma of neurons in acute murine VNO slices. The recordings were performed from the soma of neurons located in the basal zone of the VNO; therefore, we expect that most of the recorded neurons expressed receptors of the V2R family (Herrada and Dulac, 1997; Matsunami and Buck, 1997; Ryba and Tirindelli, 1997). The average zero-current membrane potential was −65 ± 6 mV (range, −84 to −53 mV), and the average input resistance was 1.3 ± 0.5 GΩ (*n* = 34). We stimulated VSNs with a time-controlled flow of diluted mouse urine, which is very rich in natural ligands for vomeronasal receptors (Moss et al., 1997; Nodari et al., 2008; Tirindelli et al., 2009; Doyle et al., 2016), and recorded the evoked spiking activity (Fig. 1*A*, green trace). On the same neuron, we used a depolarizing high-K^+^ solution stimulus as a positive control to test the neuron viability and to determine the time of arrival of the solution on the neuron (Fig. 1*A*, black trace). Moreover, as urine contains K^+^, we also performed a control by applying diluted artificial urine (Fig. 1*A*, gray trace), a solution with salt composition similar to urine (Holy et al., 2000; Wong et al., 2018; Hernandez-Clavijo et al., 2021) and discarded neurons responding to artificial urine. In this way, we verified that spiking activity in response to urine was not because of changes in ion concentration. The raster plot in Figure 1*B* shows examples of responses to 5 s urine stimulation from 10 different VSNs.

### VSNs show short-term spike frequency adaptation in response to repeated natural stimuli

**Figure 2.**
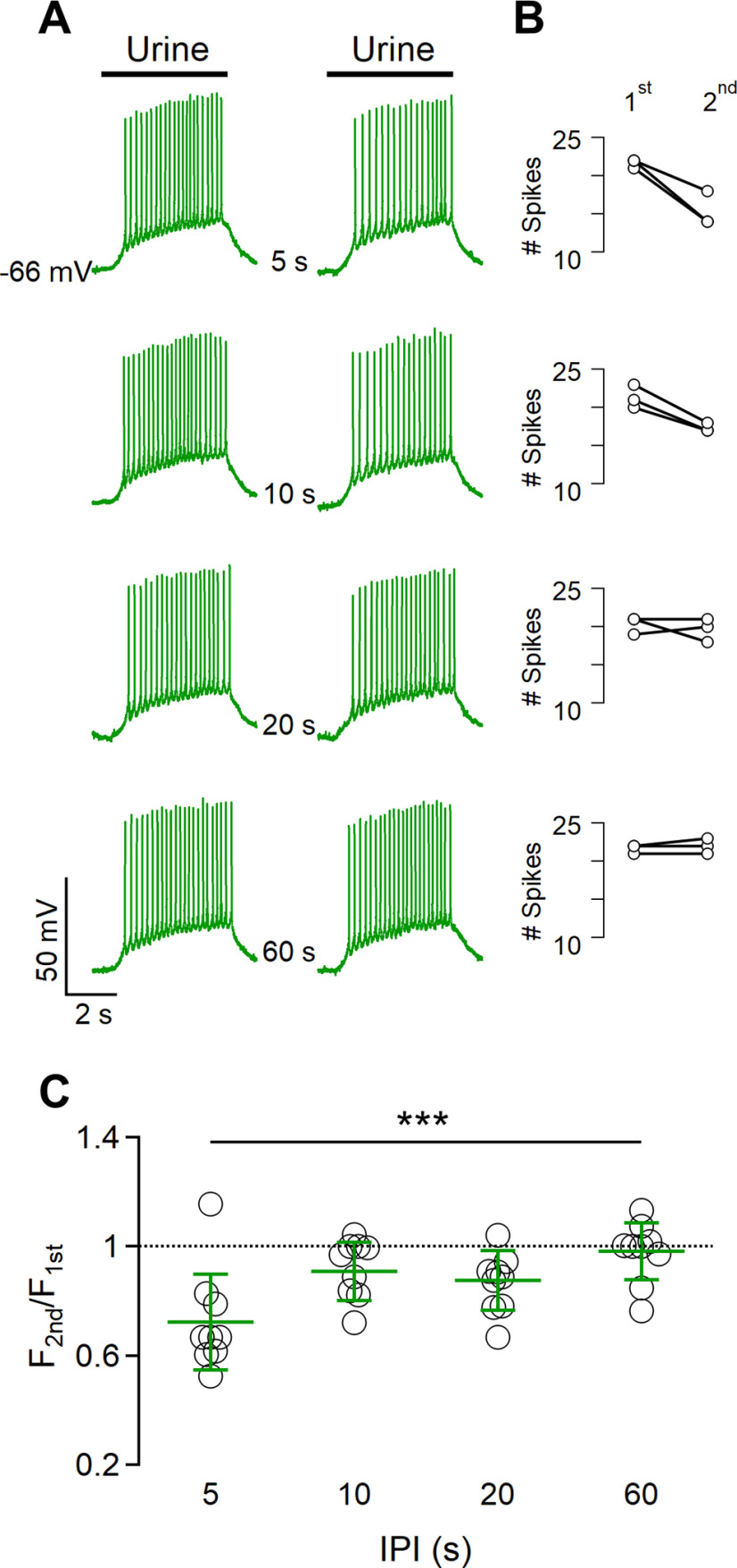
Spike frequency adaptation to repeated urine stimulations. ***A***, Representative whole-cell current-clamp recordings from a VSN repetitively stimulated with diluted urine for 5 s with increasing intervals between pulses of 5, 10, 20, or 60 s, as indicated. Black bars at the top indicate the time of urine application. ***B***, The number of spikes during the first and the second stimulation at the corresponding IPI is shown in the same row in ***A***. The paired-pulse protocol was repeated three times in the same neuron. ***C***, Scatter dot plot with the average ± SD of normalized spike frequency of the second stimulation with respect to the first stimulation for each IPI (*n* =10, Demsar’s test after Friedman test, *p* = 0.00029).

To evaluate the time course of adaptation, we first applied a 5 s urine pulse followed by a second pulse of the same duration. As we have previously reported (Wong et al., 2018), we confirm that the number of spikes during the second pulse was smaller for short IPIs but recovered toward the value of the first pulse as the IPI increased (Fig. 2*A*,*B*). The recovery time between each paired pulse was at least 2 min. To quantify sensory adaptation and compare results from several neurons, we normalized the average firing frequency during the second pulse to the value during the first one (Fig. 2*C*) and found that spike frequency was significantly different at an IPI of 5 s (average frequency ratio, 0.72 ± 0.18; *n* = 10), whereas for longer IPIs of 10, 20, and 60 s, the frequency was not significantly different.

**Figure 3.**
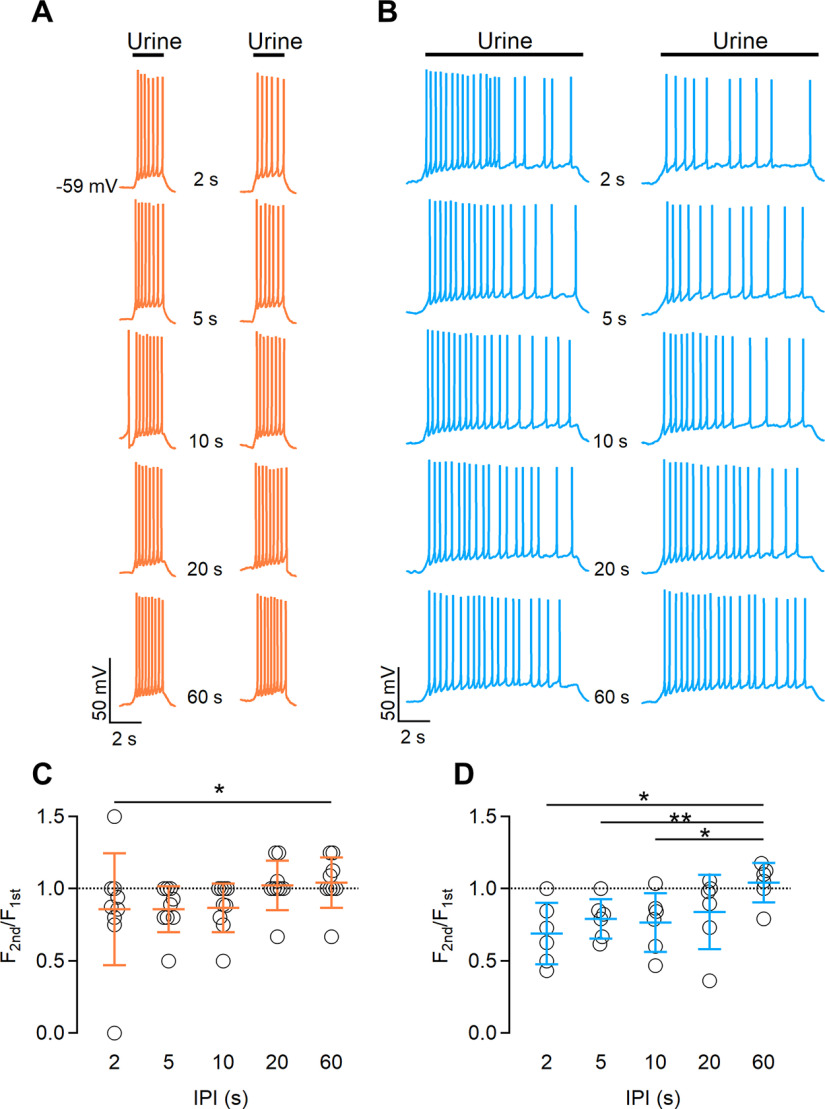
The duration of urine stimulation affects the extent of spike frequency adaptation. ***A***, ***B***, Representative whole-cell current-clamp recordings obtained stimulating VSNs with diluted urine for 2 s (***A***) or 10 s (***B***), with increasing intervals between pulses of 2, 5, 10, 20, or 60 s, as indicated. Black bars indicate the time of urine application. ***C***, ***D***, Scatter dot plots with the average ± SD of normalized spike frequencies for each IPI for urine pulses of 2 s (***C***) or 10 s (***D***; for ***C***: *n* = 9, Demsar’s test after Friedman test (*p* = 0.045 for IPI 2 s; for ***D***: *n* = 6; paired *t* test with Bonferroni correction after ANOVA for repeated measurements: *p* = 0.018 for IPI 2 s; *p* = 0.008 for IPI 5 s; and *p* = 0.036 for IPI 10 s).

To further investigate the dependence of short-term adaptation on stimulus duration, we applied urine pulses of shorter (2 s) and longer (10 s) durations than 5 s (Fig. 3*A*,*B*). We found that repetitive urine stimuli of 2 s showed adaptation only at an IPI of 2 s (Fig. 3*C*). On the other hand, paired-pulse protocols with longer urine stimulations of 10 s resulted in spike frequency adaptation when IPIs of 2, 5, and 10 s were used (average frequency ratios: 0.68 ± 0.21 for 2 s; 0.79 ± 0.14 for 5 s; and 0.76 ± 0.2 for 10 s; *n* = 9; Fig. 3*B*,*D*).

These results show that short-term adaptation in VSNs depends on the duration of urine stimulation as well as on IPI.

### VSNs show short-term spike frequency adaptation to repeated current steps

To test whether short-term adaptation is because of the adaptation of components of the signal transduction cascade and/or to inactivation of voltage-gated channels involved in action potential generation, we stimulated VSNs with current steps instead of urine, thus inducing spiking activity while bypassing the activation of the signal transduction cascade.

**Figure 4.**
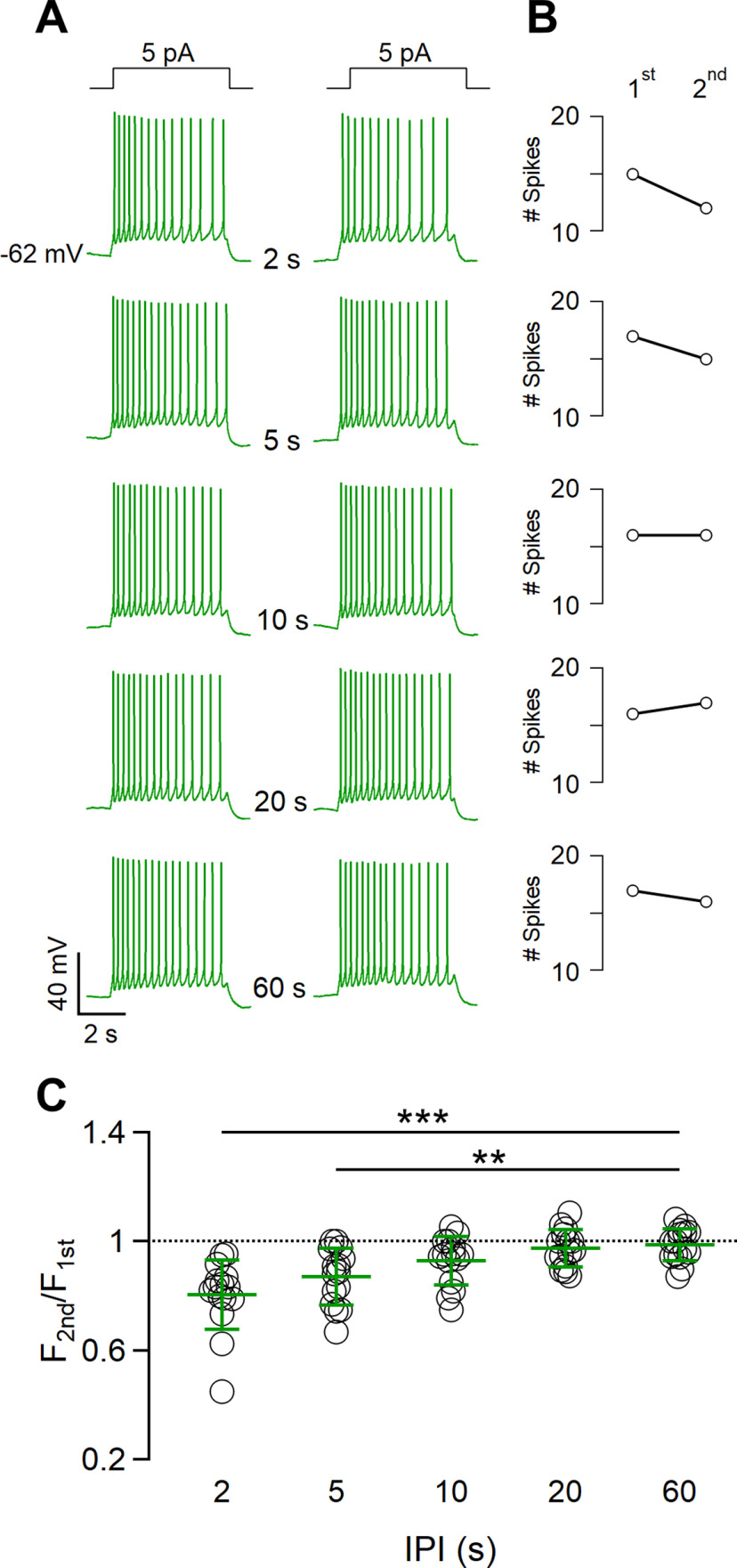
VSNs show spike frequency adaptation in response to repeated current steps. ***A***, Representative whole-cell current-clamp recordings of a VSN repetitively stimulated with a 5 pA current step for 5 s with increasing intervals between steps of 2, 5, 10, 20, or 60 s, as indicated. Steps at the top indicate the time of current injection. ***B***, Number of spikes during the first and the second stimulation at the corresponding IPI shown in the same row in ***A***. ***C***, Scatter dot plot with the average ± SD of the normalized spike frequency of the second with respect to the first stimulation for each IPI (*n* = 15; Demsar’s test after Friedman test: *p* = 7.5 * 10^–5^ for 2 s; *p* = 0.006 for 5 s).

We applied depolarizing current injections of 5 pA, a value that is sufficient to generate action potentials but does not saturate spike frequency in VSNs (Liman and Corey, 1996; Shimazaki et al., 2006). We first stimulated VSNs with a current step lasting 5 s, followed by an identical stimulus at time intervals increasing from 2 to 60 s (Fig. 4*A*,*B*). Stimulation with paired current steps produced adaptation when IPIs of 2 and 5 s were used (average frequency ratios: 0.8 ± 0.03 for 2 s; 0.87 ± 0.03 for 5 s; *n* = 15).

**Figure 5.**
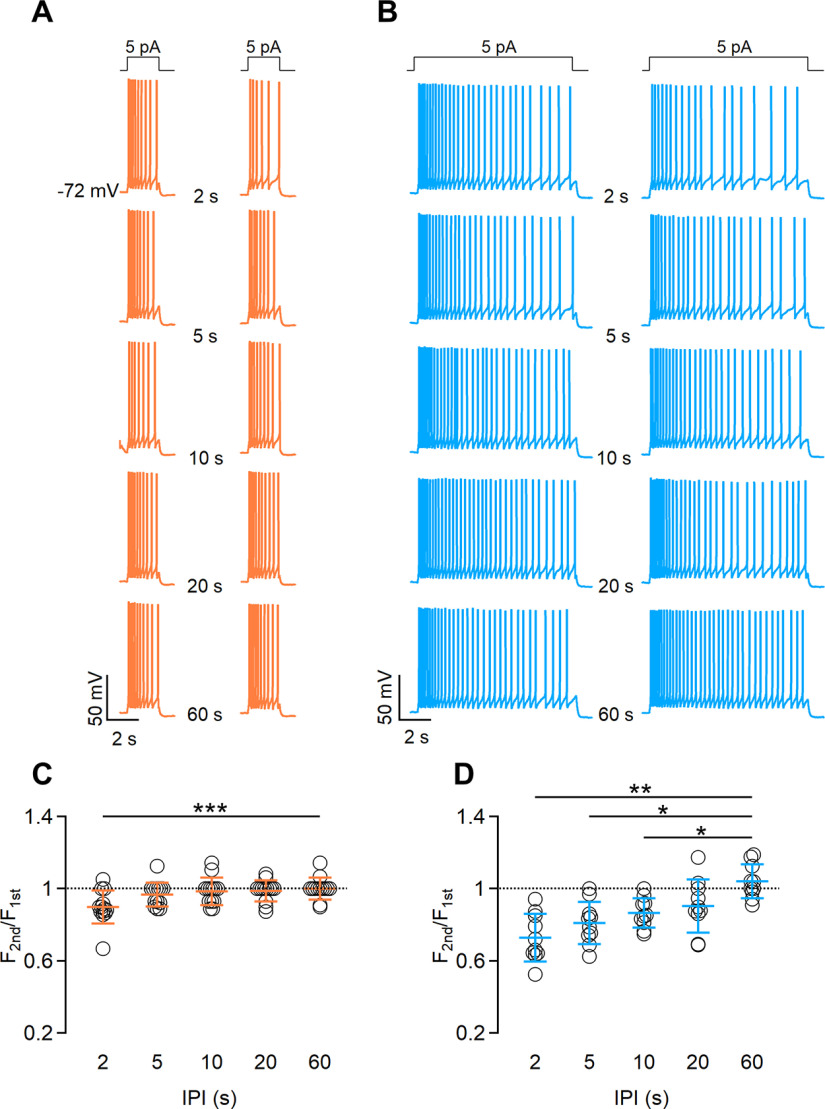
The duration of current step stimulation affects the extent of spike frequency adaptation. ***A***, ***B***, Representative whole-cell current-clamp recordings obtained stimulating VSNs with a 5 pA current step for 2 s (***A***) or 10 s (***B***), with increasing intervals between steps of 2, 5, 10, 20, or 60 s, as indicated. Steps at the top indicate the time of current injection. ***C***, ***D***, Scatter dot plots with the average ± SD of normalized spike frequencies for each IPI for current steps of 2 s (***C***) or 10 s (***D***; for ***C***: *n* = 13; Demsar’s test after Friedman test, *p* = 0.005 for IPI 2 s; for ***D***: *n* = 10; paired *t* test with Bonferroni correction after ANOVA for repeated measurements: *p* = 0.0019 for IPI 2 s; *p* = 0.01 for IPI 5 s; *p* = 0.01 for IPI 10 s).

We next examined whether the duration of a current step affects short-term adaptation by repeating the same experiments obtained with urine stimuli (Fig. 3; i.e., we applied pairs of current steps of 2 or 10 s duration at various IPIs). We found that repetitive current steps of 2 s generated short-term adaptation with an IPI of 2 s (average frequency ratio, 0.89 ± 0.09; *n* = 13), but not when longer IPIs were used (Fig. 5*A*,*C*), while current steps of 10 s caused adaptation with IPIs of 2 and 5 s (average frequency ratios: 0.79 ± 0.13 for 2 s; 0.81 ± 0.12 for 5 s; *n* = 13; Fig. 5*B*,*D*).

These data indicate that, at least partially, ion channels involved in the generation of action potentials, independent of signal transduction cascade components, might contribute to the development of short-term spike frequency adaptation in VSNs.

### Phase–plane plot analysis of action potentials during repetitive stimulations

**Figure 6.**
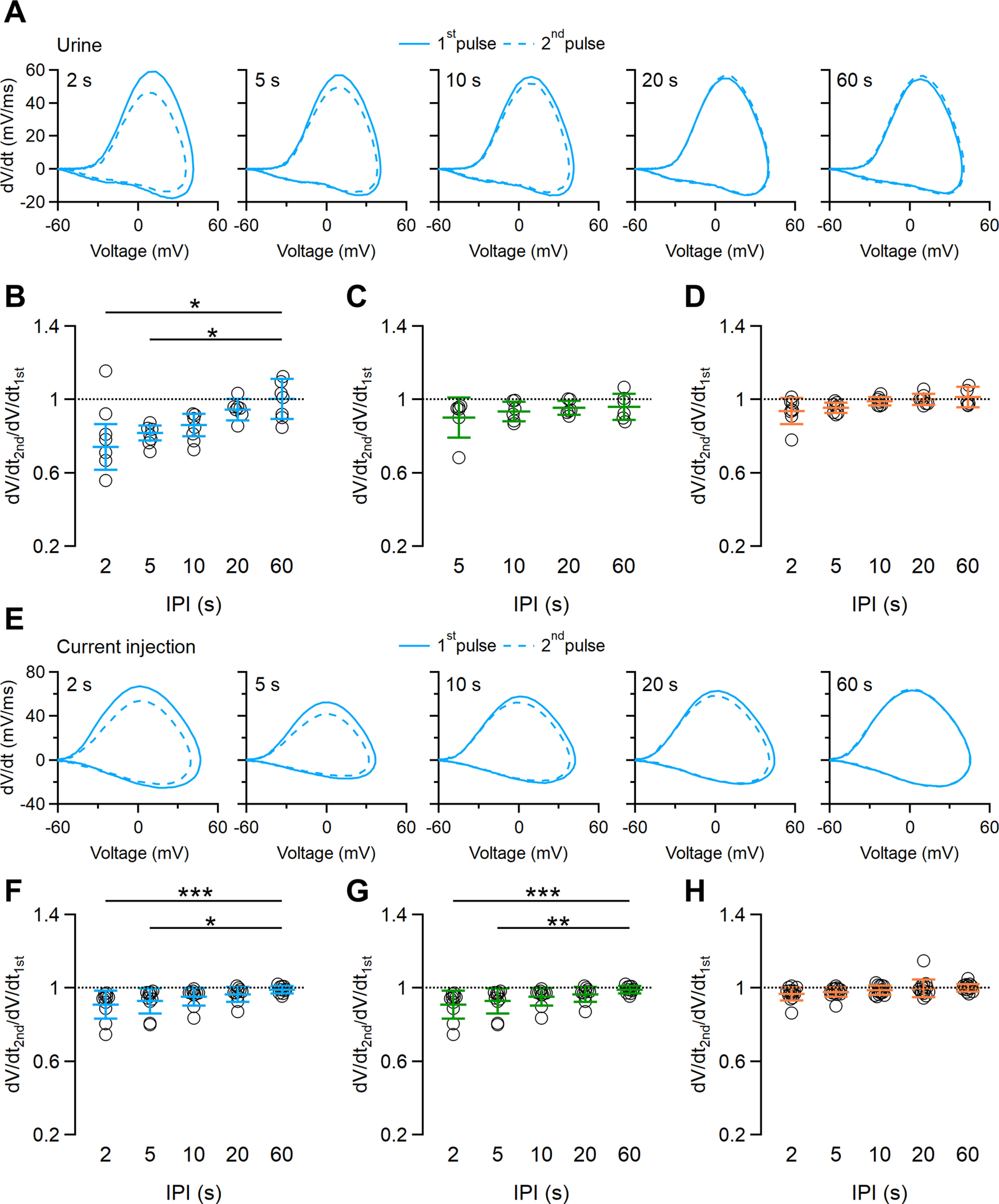
Phase–plane plot analysis of action potentials from repeated stimulations. ***A***, Phase–plane plots of the first action potential in response to a repeated urine stimulation of 10 s duration with increasing IPIs ranging from 2 to 60 s, as indicated. Continuous and dashed lines were calculated from the first action potential of the first and second urine pulses, respectively. ***B–D***, Scatter dot plots with the average ± SD of the ratio between the maximal dV/dt values of the first action potentials at each IPI. Urine pulse durations of 10 s (***B***), 5 s (***C***), and 2 s (***D***; for ***B***: *n* = 6; Demsar’s test after Friedman test: *p* = 0.0021 for IPI 2 s; *p* = 0.0041 for IPI 5 s; for ***C***: *n* = 6; Friedman test, *p* = 0.89; for ***D***: *n* = 9; Friedman test, *p* = 0.051). ***E***, Phase–plane plots of the first action potential in response to a repeated 5 pA current step of 10 s duration with increasing IPIs ranging from 2 to 60 s, as indicated. Continuous and dashed lines were calculated from the first action potential of the first and second current steps, respectively. ***F–H***, Scatter dot plots with the average ± SD of the ratio between the maximal dV/dt values of the first action potentials at each IPI. Current step durations of 10 s (***F***), 5 s (***G***), and 2 s (***H***; for ***F***: *n* = 8; Demsar’s test after Friedman test: *p* = 0.00054 for IPI 2 s; *p* = 0.018 for IPI 5 s; for ***H***: *n* = 14; Friedman test, *p* = 0.126).

To obtain further information about ion channels involved in spike frequency adaptation, we analyzed the first action potentials during the first and second paired pulses by using phase–plane plot analysis. In this type of analysis, the changes of membrane potential with time (dV/dt) are plotted as a function of membrane potential, and each action potential is represented by a loop, with the upper and lower parts representing the depolarization and repolarization phases, respectively (Jenerick, 1963; Naundorf et al., 2006; Bean, 2007). The maximal value of dV/dt is the maximal rising slope of the depolarization phase of the action potential, which is related to the amplitude of Na^+^ currents and, consequently, to the availability of Na^+^ channels (Bean, 2007). In Figure 6*A*, we superimposed phase–plane plots calculated from the first action potential evoked by 10 s urine pulses at different IPIs. At 2 s IPI, the maximal value of dV/dt was clearly lower for the first action potential evocked by the second urine pulse (46 mV/ms; Fig. 6*A*, dashed line) compared with the one evoked by the first urine pulse (59 mV/ms; Fig. 6*A*, continuous line), while increasing IPIs narrowed the differences between the maximal values of dV/dt between the first action potentials of the repeated urine pulses. On average, the ratio between maximal values of dV/dt was significantly different at 2 and 5 s IPIs (0.74 ± 0.12 for 2 s; 0.81 ± 0.04 for 5 s; *n* = 6; Fig. 6*B*). The reduction of the maximal dV/dt value indicates a contribution of Na^+^ channels to spike frequency adaptation to urine (Fig. 6*B*). We did not measure any significant difference in maximal dV/dt for shorter urine paired pulses of 2 and 5 s (Fig. 6*C*,*D*).

We performed the same analysis when VSNs were stimulated with paired current steps as in Figures 4 and 5. In a way similar to that of urine stimulation, phase–plane plots for current paired pulses of 10 s show a clear difference in maximal dV/dt during a second stimulation when short IPIs (2 and 5 s) were used, and no differences were observed at longer IPIs (Fig. 6*F*). Current pulses of 5 s also displayed some difference in maximal dV/dt (Fig. 6*G*), while for current pulses of 2 s we did not measure any significant difference in maximal dV/dt (Fig. 6*H*).

Together, these data indicate that voltage-gated Na^+^ channels involved in action potential generation contribute to short-term spike frequency adaptation measured with paired urine pulses.

### Inactivation properties of voltage-gated Na^+^ currents in VSNs

**Figure 7.**
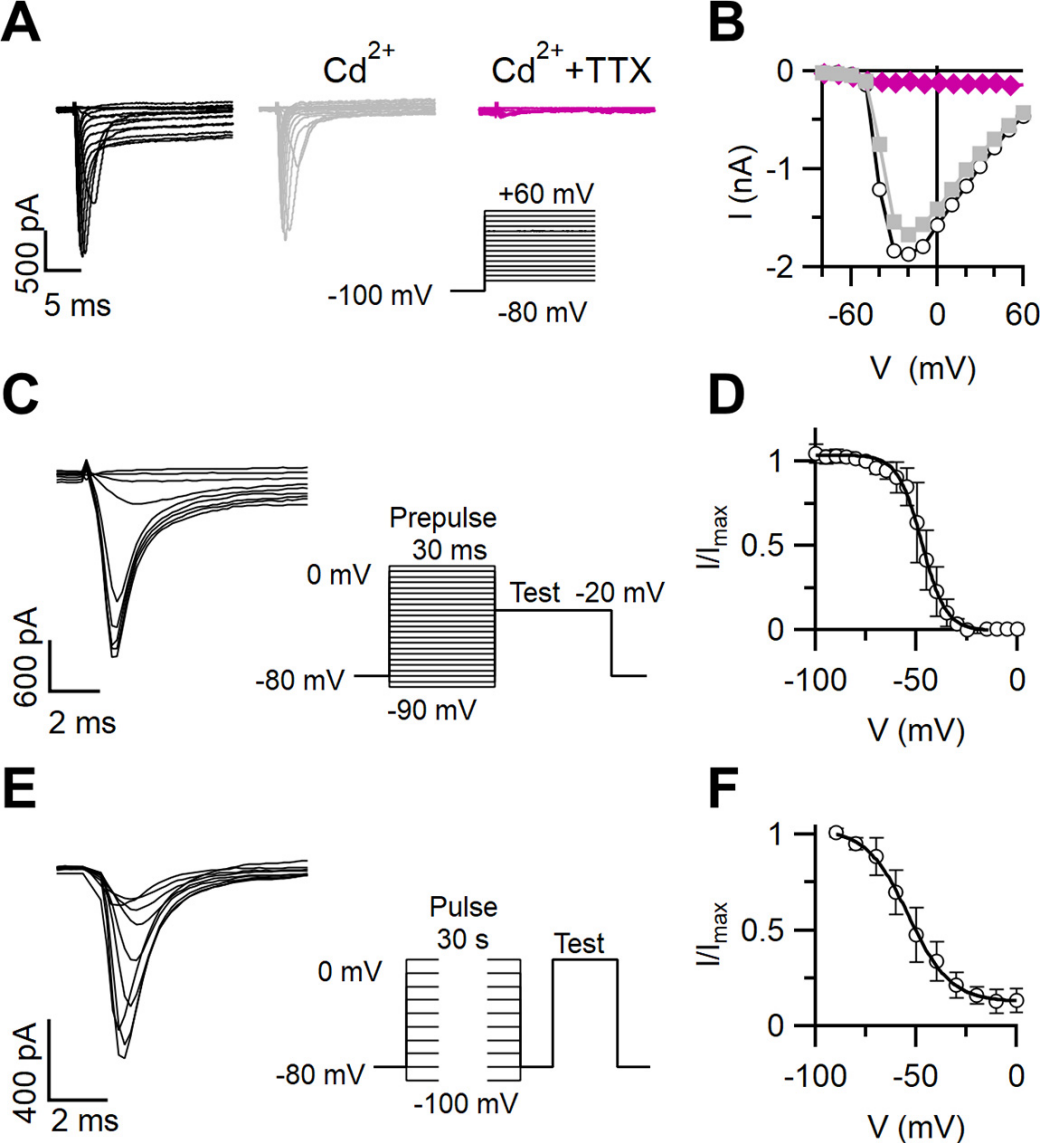
Inactivation of Na^+^ currents in VSNs. ***A***, Families of whole-cell voltage-gated inward currents recorded from a VSN elicited by voltage steps from −80 to +60 mV with 10 mV increments from a holding potential of −100 mV. The patch pipette contained a Cs^+^-based intracellular solution. The neuron was exposed successively to bath solutions containing 100 μm Cd^2+^ and 100 μm Cd^2+^ plus 2 μm TTX, as indicated. ***B***, Current–voltage relations for the peak inward currents from the recordings in ***A***. ***C***, ***E***, Current recordings from the same VSN shown in ***A*** in response to stimulation protocols to measure inactivation in the presence of 100 μm Cd^2+^. A test pulse was preceded by a prepulse at the indicated voltages of 30 ms (***C***) or 30 s (***E***) duration to measure fast and slow inactivation, respectively. In ***C***, one every two traces is shown. ***D***, ***F***, Normalized currents versus membrane potential from the experiments shown in ***C*** and ***E*** were fitted with a Boltzmann equation with *V*_half_ = −46.7 ± 1.6 mV and *k *=* *4.9 ± 1.3 mV (*n* = 8) for fast inactivation (***D***) and *V*_half_ = −50.6 ± 2.4 mV and *k *=* *8.7 ± 1.3 mV (*n* = 6) and asymptotic value of *A* = 0.18 for slow inactivation (***F***).

To measure the inactivation properties of Na^+^ channels in VSNs, we recorded inward currents from individual VSNs in the whole-cell voltage-clamp configuration using a Cs^+^-based intracellular solution to block outward K^+^ currents. Representative inward currents generated in response to a series of step depolarizations from a holding potential of −100 mV are shown in Figure 7*A*. As both Na^+^ and Ca^2+^ currents may contribute to inward currents in VSNs (Liman and Corey, 1996; Ukhanov et al., 2007), we added 100 μm Cd^2+^ to the external solution to block Ca^2+^ currents. The remaining inward currents were abolished by the addition of 2 μm TTX, indicating that they were mainly carried by TTX-sensitive Na^+^ channels (Fig. 7*A*,*B*). On average, the Na^+^ current was 83 ± 2% (*n* = 8) of the total inward current at −20 mV.

To measure the voltage dependence of steady-state fast and slow inactivation of Na^+^ channels, we used typical voltage protocols composed of prepulses and a test pulse to evaluate the noninactivated fraction of Na^+^ channels (all recordings were performed in the presence of 100 μm Cd^2+^ in the bath). For fast inactivation, we used prepulses of 30 ms at voltages from −90 to 0 mV, followed by a test potential of 30 ms at −20 mV (Fig. 7*C*). Normalized currents plotted as a function of the prepulse potential were fitted by the Boltzmann equation (Fig. 7*D*), and data from several neurons gave the following parameters: *V*_half_ = −46.7 ± 1.6 mV; and *k *=* *4.9 ± 1.3 mV (*n* = 8). As Na^+^ channels can also enter a slow inactivated state, which might be relevant to our study of adaptation, we induced slow inactivation by applying prepulses of 30 s (Fig. 7*E*) at voltages from −90 to 0 mV, followed by a short (20 ms) hyperpolarizing step (to separate fast from slow inactivation) and by a test potential (30 ms) at 0 mV (Fig. 7*E*). The fit with a Boltzmann equation of the normalized current versus the prepulse potential (Fig. 7*F*) resulted in *V*_half_ = −50.6 ± 2.4 mV and *k *=* *8.7 ± 1.3 mV (*n* = 6).

**Figure 8.**
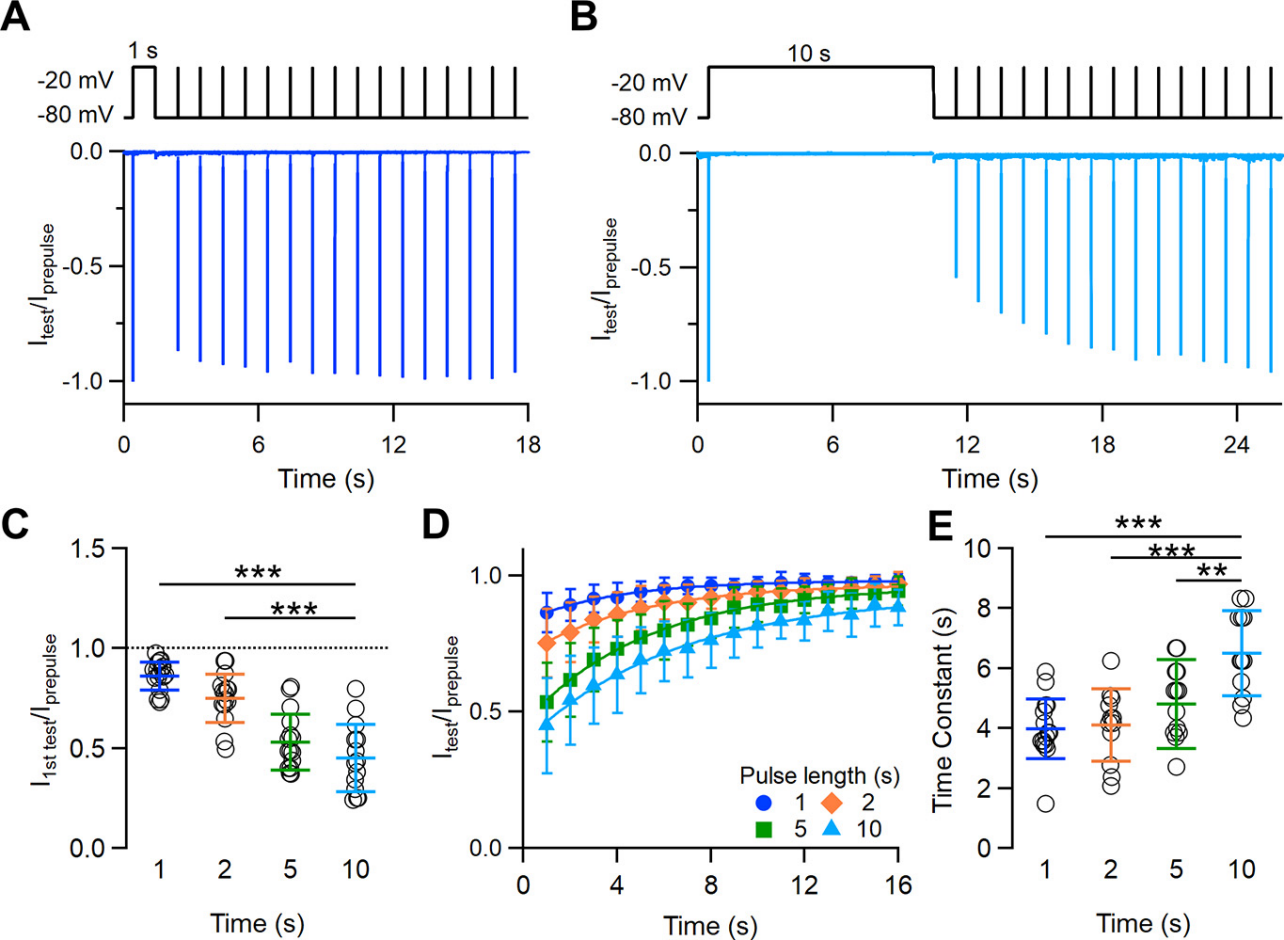
Time course of recovery of Na^+^ channels from inactivation induced by depolarization steps varying from 1 to 10 s duration. ***A***, ***B***, Representative whole-cell voltage-clamp recordings of Na^+^ currents in a VSN in the presence of 100 μm Cd^2+^. Currents were elicited by a paired-pulse protocol consisting of a depolarization prepulse from −80 to −20 mV of 1 s (***A***) or 10 s (***B***) duration followed by a short (10 ms) test pulse at increasing recovery intervals ranging from 1 to 15 s. The holding potential was −80 mV. For each IPI, the peak current measured at the test pulse was normalized to the prepulse peak current and superimposed. ***C***, Scatter dot plots with the average ± SD of the normalized peak currents measured at 1 s after the prepulse of the indicated duration (*t* test with Bonferroni correction after ANOVA for repeated measurements: *p* =1.7 * 10^−6^ for 1 s; *p* = 1.97 * 10^−6^ for 2 s; *p* = 0.22 for 5 s; *n* = 14–17). ***D***, Recovery from inactivation as a function of IPIs at the indicated prepulse duration. ***E***, Scatter dot plot with the average ± SD of the time constants (τ) of recovery from slow inactivation (Dunnett’s test after ANOVA: *p* = 9.11 * 10^−6^ for 1 s; *p* = 3.97 * 10^−5^ for 2 s; *p* = 0.0045 for 5 s; *n* = 12–15).

As Na^+^ channels may enter a state of slow inactivation on long depolarizations from which they recover only after several seconds, we measured the time course of recovery of Na^+^ channels in VSNs with a typical paired-pulse protocol with depolarizing prepulses varying from 1 to 10 s in duration. A depolarizing prepulse of a selected duration was given from a holding potential of −80 to −20 mV to induce inactivation, followed by a second pulse (test) with IPIs varying from 1 to 15 s (Fig. 8*A*,*B*, top line). The neuron was kept at −80 mV during each IPI. Moreover, to allow a complete recovery of the Na^+^ current, the time between each consecutive paired pulse was at least 1 min. The peak current measured 1 s after each prepulse (duration, 1–10 s) was smaller than that measured at the prepulse; but, as IPI increased, more channels recovered from inactivation and the peak current produced by the second pulse gradually recovered toward the initial value. Figure 8, *A* and *B*, shows the superimposition of ratios of the peak currents evoked by the second and first pulses at IPIs from 1 to 15 s for depolarizing prepulses of 1 or 10 s, respectively. The first test pulse, applied 1 s after the depolarizing prepulse, produced a Na^+^ current of decreasing amplitude as the prepulse duration increased (average current ratios: 0.86 ± 0.07 for 1 s; 0.75 ± 0.12 for 2 s; 0.53 ± 0.14 for 5 s; 0.45 ± 0.17 for 10 s; *n* = 14–17; Fig. 8*A–C*). The recovery of the Na^+^ current toward its initial amplitude was slower for longer prepulse durations; indeed, the fit of the data with an exponential function gave time constants significantly higher for longer prepulses (4.0 ± 1.0 s for 1 s; 4.1 ± 1.2 for 2 s; 4.9 ± 1.2 s for 5 s; 6.5 ± 1.4 for 10 s; *n* = 12–15; Fig. 8*D*,*E*).

These data show that voltage-gated Na^+^ channels in VSNs present slow inactivation and that recovery from slow inactivation requires several seconds and depends on the duration of the inactivation step. The time scale of seconds for recovery from slow inactivation is similar to what was measured during short-term adaptation protocols, suggesting that the slow recovery of Na^+^ channels from inactivation partially contributes to short-term spike frequency adaptation observed in VSNs.

## Discussion

In this study, we have shown that VSNs undergo spike frequency short-term adaptation when stimulated with repetitive pulses of natural stimuli in current-clamp whole-cell recordings. This type of protocol resembles the temporal arrival of stimuli to the VNO when animals are actively sampling the environment for chemical cues. Moreover, we have shown that bypassing signal transduction activation with repetitive current injections still produced spike frequency adaptation and that slow inactivation of Na^+^ channels is one of the relevant molecular mechanisms involved in short-term adaptation in VSNs.

Our study confirms and significantly extends a previous study that revealed adaptation of VSNs when stimulated with paired-pulse protocols. Indeed, Wong et al. (2018) used *in situ* Ca^2+^ imaging and extracellular electrophysiological recordings to measure responses to various chemical cues (diluted urine and bile acid ligands) and found evidence for short- and long-term adaptation in VSNs stimulated with repetitive pulses of natural stimuli. Here, we used current-clamp whole-cell recordings to measure responses to repeated identical pulses of diluted urine from 2 to 10 s in duration, and measured spike frequency adaptation that depended on the time interval between pulses and gradually recovered as the IPI increased into the range of 2–30 s. Moreover, we found that increasing the duration of paired stimuli produced a more effective adaptation, thus demonstrating that short-term adaptation depends on the pulse duration and the interval between stimulations.

The use of whole-cell instead of loose-patch recordings, as in the study by Wong et al. (2018), allowed us to bypass the transduction cascade to measure the contribution of voltage-gated channels to spike frequency adaptation. Unexpectedly, we found that spike frequency adaptation was still present when VSNs were stimulated by repeated current step injections, independent of signal transduction activation. Thus, we used phase–plane plot analysis to compare the action potential dynamics of the first action potentials evoked by paired-pulse stimulation both with natural stimuli and with current injections. We found a significant difference in the maximal value of dV/dt of the first action potential elicited by the second compared with the first stimulus for 10 s paired pulses. As in this type of analysis the maximal value of dV/dt is directly proportional to the availability of voltage-gated Na^+^ channels (Bean, 2007), these results suggested a contribution of Na^+^ channels to short-term adaptation.

A previous study by Spehr et al. (2009) showed that VSNs undergo spike frequency adaptation by measuring current-clamp responses to urine paired pulses of 20 s duration separated by an IPI of 30 s and that this form of adaptation was mainly caused by Ca^2+^-calmodulin feedback inhibition of the TRPC2 transduction channel. Moreover, the authors did not find any spike frequency adaptation when VSNs responded to paired current steps of 2 pA lasting 20 s and separated by an IPI of 30 s, thus excluding a contribution of voltage-gated channels. It is important to note that time scales are different in our experiments, and therefore the results cannot be directly compared. Indeed, we used urine pulses from 2 to 10 s, shorter than the 20 s pulses used by Spehr et al. (2009), and we found that spike frequency adaptation was not present at the time interval of 30 s. Thus, our results are not in contrast with those of Spehr et al. (2009) and do not exclude a contribution to adaptation of the Ca^2+^-calmodulin feedback, but rather introduce Na^+^ channels as new players in short-term spike frequency adaptation.

To evaluate the relative importance of sensory transduction modulation and the slow inactivation of the Na^+^ channel determining the VSN adaptation, it would be necessary to stimulate the VSNs with a different current amplitude to generate action potential firing similar to that induced by pheromone application. Unfortunately, the pattern of firing induced by urine is very heterogeneous (Fig. 1) precluding a precise quantification of the results.

To better understand the mechanisms of action of Na^+^ channels on the measured spike frequency adaptation, we analyzed their properties in VSNs. Voltage-gated Na^+^ channels undergo rapid activation to initiate the rising phase of action potential, followed by fast and slow inactivation processes. While fast inactivation attenuates inward Na^+^ conductance on the millisecond time scale, slow inactivation occurs when prolonged or high-frequency depolarizations over the span of seconds reduce the number of channels available to provide excitatory inward currents (Silva, 2014). Slow inactivation of voltage-gated Na^+^ channels is well known to play a role in controlling membrane excitability, firing properties, and spike frequency adaptation in the nervous system (Fleidervish et al., 1996; Toib et al., 1998). We investigated the inactivation properties of Na^+^ currents recorded in VSNs with paired-pulse protocols and measured a slow inactivation that required several seconds to recover and was dependent on the duration of the inactivation step. The time scale of recovery from slow inactivation was similar to that measured for short-term adaptation induced by urine and current step stimuli, indicating that the slow recovery from inactivation contributes to spike frequency adaptation to paired-pulse protocols.

A previous study investigated in detail the Na^+^ currents in subpopulations of VSNs specifically expressing V1Rb2 (apical zone) or V2R1b (basal zone; Ukhanov et al., 2007). Although it is difficult to generalize the results found in V1Rb2-expressing and V2R1b-expressing cells to the whole population of apical and basal VSNs, respectively, the parameter of the Boltzmann equation we found for fast inactivation is indeed in good agreement with the values reported by Ukhanov et al. (2007) for the basal neurons V2R1b. Indeed, the *V*_half_ of fast inactivation measured by us was −46.7 mV, more similar to −53.5 mV in V2R1b than to −65.7 mV in V1Rb2.

Several Na^+^ channel isoforms have been found to be expressed in VSNs either at mRNA level by RT-PCR or by immunohistochemistry, as follows: Na_v_1.1, Na_v_1.2, Na_v_1.3, Na_v_1.6, and Na_v_1.7. They differ in level of expression and are localized in different neuronal compartments (Fieni et al., 2003; Rupasinghe et al., 2012; Ibarra-Soria et al., 2014; Bolz et al., 2017). For example, Na_v_1.3 and Na_v_1.7 are highly expressed and mainly located on axons, while Na_v_1.2 and Na_v_1.6 were found in the soma of VSNs (Bolz et al., 2017).- Thus, a combination of these Na^+^ channel isoforms and their associated β-subunits is likely to contribute to the Na^+^ currents and the generation of action potentials in VSNs.

Slow inactivation of Na^+^ channels has been associated with activity-dependent modulation of neuronal excitability in several systems. For example, in neocortical pyramidal cells, Na^+^ channel slow inactivation is associated with cumulative adaptation of spike firing caused by prolonged and repetitive depolarizing stimuli and with a slowing of the rising phase of the action potentials (Fleidervish et al., 1996). In hippocampus CA1 pyramidal neurons, cumulative inactivation is involved in regulating back-propagating action potential amplitude and can influence dendritic excitation (Jung et al., 1997; Mickus et al., 1999). Interestingly, slow inactivation of Na^+^ channels has been associated with an adaptation process in the visual sensory system in the salamander, where it is involved in light temporal contrast adaptation in retinal ganglion cells (Kim and Rieke, 2003). Increasing contrast causes an increase in input current variance and consequently of the spike rate that in turn reduces Na^+^ channels availability through slow inactivation (Kim and Rieke, 2003).

In summary, our work confirms and extends previous evidence of short-term adaptation in VSNs when stimulated with paired pulses of natural stimuli (Spehr et al., 2009; Wong et al., 2018) and demonstrates the contribution of a new molecular player by showing that slow inactivation of Na^+^ channels is an important component of short-term adaptation to natural stimuli.

## Synthesis

Reviewing Editor: Christina Zelano, Northwestern University - Chicago Decisions are customarily a result of the Reviewing Editor and the peer reviewers coming together and discussing their recommendations until a consensus is reached. When revisions are invited, a fact-based synthesis statement explaining their decision and outlining what is needed to prepare a revision will be listed below. The following reviewer(s) agreed to reveal their identity: Roberto Tirindelli, Haiqing Zhao.

The reviewers and I agree that this manuscript expands our understanding of sensory adaptation and makes an important contribution to the field. However, both reviewers raised important questions that need to be addressed before the manuscript can be accepted. See their comments below:

Reviewer 1:

In general, this paper addresses an important issue in chemosensory research, namely the mechanism of sensory adaptation to persistent stimulation. Here, the authors studied the mechanism underlying adaptation in the vomeronasal sensory neurons (VSNs), which detect pheromones. They showed that the inactivation properties of the voltage-gated sodium current (INa) might play a role in the reduced response to repeated stimulation. As a whole, the study is well conceived and the paper is well written. Nonetheless, I have some points of concerns that should be adequately addressed.

1. VSNs comprise two main groups of cells, apical and basal neurons. It is well known that excitability properties, such as repetitive firing, differ among these neurons. In addition, INa exhibits different properties, including steady-state inactivation. Since the authors used a slice preparation, it should be easy to establish whether they recorded from apical or basal VSNs. This missing information downgrades the overall paper.

2. The lack of information on the VSN types they recorded from hampers the possibility to compare their data with published ones. For example, it would be nice to know whether the parameters of the Boltzmann equations for fast inactivation are in agreement with those found by others.

3. Current traces shown in Figure 7C exhibit poor space clamping. This inevitably affects the Boltzmann plots. Long pre-pulses tend to shift the inactivation curve to more negative membrane potentials. Indeed, they found that Vhalf for slow inactivation was more negative than Vhalf for fast inactivation. Were these values significantly different? I some doubts on it given the poor recording conditions.

4. The authors missed to satisfactorily discuss their data in the context of the current knowledge. For example, Ukhanov et al. (2007) studied in detail the excitability and the ion currents in VSNs, but I failed to find any reference to this paper in the Discussion.

5. It is not clear what the authors mean with “resting membrane potential” (line 154). Is it the zero-current potential? I would not use the term “resting potential” because any measurement with the patch-clamp technique is inevitably affected by the shunt seal resistance.

6. Although the authors showed many current clamp recordings with repetitive firing (Fig. 1 to 5), I failed to find any indication of the membrane potential of neurons when they applied the stimulus. This information should be added.

7. Figure 7 D and F: replace single values with means {plus minus} SD.

8. Line 175: n = 10, whereas in the fig. 2 legend n = 9. Please correct.

9. Line 185: “show-term spike” Please correct.

Reviewer 2:

In this manuscript, the authors investigate short-term spike adaptation of vomeronasal neurons (VSNs). They show that VSNs display short-term spike adaptation using whole-cell patch-clamp recording and paired-pulse stimulation protocol. The authors first used paired pulses of diluted urine and found that VSNs exhibited spike adaptation to repeated stimulations of natural stimulants. The result confirmed the observation of earlier publications. They then investigated action potential generation caused by repeated injections of the depolarizing current, which bypassed the sensory transduction step. Contrary to a previous report, the authors found that repeated current injections were sufficient to cause spike adaption in VSNs when the interpulse intervals were short. The authors further analyzed the inactivation properties of voltage-gated Na+ channels and demonstrated that voltage-gated Na+ channels in VSNs exhibited slow inactivation. The authors concluded that the slow inactivation of Na+ channels contributed to the short-term spike adaptation of VSNs to repeated stimulations. The experiments presented in the manuscript are well-done, and the analysis are adequate. The conclusion is convincingly supported by the results.

As the authors also implied in the manuscripts (Line 186), an obvious question here is the relative contribution of the transduction-originated adaptation and Na channel-originated adaptation to the spike adaption. I do have a couple of questions regarding the experimental design that I would like to hear the authors' thoughts on. First, in the case of the current injection experiments (Figs 4 and 5), I understand it is easier to use a current of 5 pA to generate spikes, but would it be more informative to use a current that would generate a comparable number of spikes (as the diluted urine would (e.g., some 20 spikes/5 sec)? Although each VSN would need a different amount of current, current injection in such a manner should make it possible to evaluate the relative importance of the transduction-originated spike adaptation vs. the Na channel inactivation-originated adaption. Such analysis would make this paper much more impactful. The authors should at least discuss this question in the Discussion. Also, do the VSNs used for the current injection experiments pass the Fig 1 test? Second, in the experiments characterizing the slow inactivation of Na+ channels (Fig 7), Fig 7F curve should contain both the fast inactivation and the slow inactivation components. Why did the test pulse in Figure 7E set to 0 mV instead of -20 mV? If it were set at -20 mV, Fig 7F curve would be able to compare directly with Fig 7D curve, and the difference would indicate the slow inactivation. With the curve obtained at 0 mV, how could the authors know that the channel underwent slow inactivation?

There are a few minor errors.

Line 155, “average input resistance was 1.3 {plus minus} 0.5 (n = 34)”. The resistance unit is missing.

Line 185, “show-term” should be “short-term"

Figure 7E, what does the “-100 mV” mean?

## Author Response

To Dr. Christina Zelano, Reviewing Editor of eNeuro

Revision of the manuscript eN-NWR-0471-21 “Slow inactivation of sodium channels contributes to short-term adaptation in vomeronasal sensory neurons”.

We thank the Reviewing Editor and the two Reviewers for their careful reading and constructive comments on our manuscript. We believe that the revised manuscript has been improved by taking into account all the comments. We address point by point the specific comments below.

Reviewer 1:

In general, this paper addresses an important issue in chemosensory research, namely the mechanism of sensory adaptation to persistent stimulation. Here, the authors studied the mechanism underlying adaptation in the vomeronasal sensory neurons (VSNs), which detect pheromones. They showed that the inactivation properties of the voltage-gated sodium current (INa) might play a role in the reduced response to repeated stimulation. As a whole, the study is well conceived and the paper is well written. Nonetheless, I have some points of concerns that should be adequately addressed.

We thank the Reviewer for positive comments

1. VSNs comprise two main groups of cells, apical and basal neurons. It is well known that excitability properties, such as repetitive firing, differ among these neurons. In addition, INa exhibits different properties, including steady-state inactivation. Since the authors used a slice preparation, it should be easy to establish whether they recorded from apical or basal VSNs. This missing information downgrades the overall paper.

Please see the answer below to point 2

2. The lack of information on the VSN types they recorded from hampers the possibility to compare their data with published ones. For example, it would be nice to know whether the parameters of the Boltzmann equations for fast inactivation are in agreement with those found by others.

Localization of recorded VSNs inside the slice can be an indication of the type of cell recorded, but without the use of a genetically encoded label we cannot be sure what type of cells we are recording from. However, since we recorded from soma of cells in the basal region of the VNO, we expect that most recorded neurons are basal VSNs, as we reported at lines 151-153, page 4.

Ukhanov et al. (2007) used one subpopulation of apical VSNs specifically expressing V1Rb2 receptor, and one subpopulation of basal VSNs specifically expressing V2R1b receptor. It is difficult to generalize the results found in V1Rb2-expressing and V2R1b-expressing cells to the whole population of apical and basal VSNs, respectively. However, the parameter of the Boltzmann equation we found for fast inactivation are indeed in good agreement with the values reported by Ukhanov et al. (2007) for the basal neurons V2R1b. We discussed it at page 10, lines 338-344.

3. Current traces shown in Figure 7C exhibit poor space clamping. This inevitably affects the Boltzmann plots. Long pre-pulses tend to shift the inactivation curve to more negative membrane potentials. Indeed, they found that Vhalf for slow inactivation was more negative than Vhalf for fast inactivation. Were these values significantly different? I some doubts on it given the poor recording conditions.

We agree with the reviewer that the space clamp is poor, due to the high current density and to spatial distribution of Na channels in different compartments (soma, dendrite and knob and axon). We could not use an active series resistance and capacitive compensation due to non-linearity of the system and to the presence of oscillations that damage the neuron. Due to the poor clamp conditions and to the different slope of the inactivating curves it is difficult to drive conclusions about the significance of the difference of the Vhalf for fast and slow inactivation.

4. The authors missed to satisfactorily discuss their data in the context of the current knowledge. For example, Ukhanov et al. (2007) studied in detail the excitability and the ion currents in VSNs, but I failed to find any reference to this paper in the Discussion.

As reported for point 1 and 2, we compared our results with those reported by Ukhanov et al. (2007) at line XXX, page 10 in the Discussion.

5. It is not clear what the authors mean with “resting membrane potential” (line 154). Is it the zero-current potential? I would not use the term “resting potential” because any measurement with the patch-clamp technique is inevitably affected by the shunt seal resistance.

We changed in accordance with Reviewer suggestion.

6. Although the authors showed many current clamp recordings with repetitive firing (Fig. 1 to 5), I failed to find any indication of the membrane potential of neurons when they applied the stimulus. This information should be added.

In Figures 1-5, we added the values of zero-current potentials.

7. Figure 7 D and F: replace single values with means {plus minus} SD.

We changed the Figure 7D and F with the means and SD values.

8. Line 175: n = 10, whereas in the fig. 2 legend n = 9. Please correct.

We corrected the value in the legend

9. Line 185: “show-term spike” Please correct.

We corrected the typo.

Reviewer 2:

In this manuscript, the authors investigate short-term spike adaptation of vomeronasal neurons (VSNs). They show that VSNs display short-term spike adaptation using whole-cell patch-clamp recording and paired-pulse stimulation protocol. The authors first used paired pulses of diluted urine and found that VSNs exhibited spike adaptation to repeated stimulations of natural stimulants. The result confirmed the observation of earlier publications. They then investigated action potential generation caused by repeated injections of the depolarizing current, which bypassed the sensory transduction step. Contrary to a previous report, the authors found that repeated current injections were sufficient to cause spike adaption in VSNs when the interpulse intervals were short. The authors further analyzed the inactivation properties of voltage-gated Na+ channels and demonstrated that voltage-gated Na+ channels in VSNs exhibited slow inactivation. The authors concluded that the slow inactivation of Na+ channels contributed to the short-term spike adaptation of VSNs to repeated stimulations. The experiments presented in the manuscript are well-done, and the analysis are adequate. The conclusion is convincingly supported by the results.

As the authors also implied in the manuscripts (Line 186), an obvious question here is the relative contribution of the transduction-originated adaptation and Na channel-originated adaptation to the spike adaption. I do have a couple of questions regarding the experimental design that I would like to hear the authors’ thoughts on.

First, in the case of the current injection experiments (Figs 4 and 5), I understand it is easier to use a current of 5 pA to generate spikes, but would it be more informative to use a current that would generate a comparable number of spikes (as the diluted urine would (e.g., some 20 spikes/5 sec)? Although each VSN would need a different amount of current, current injection in such a manner should make it possible to evaluate the relative importance of the transduction-originated spike adaptation vs. the Na channel inactivation-originated adaption. Such analysis would make this paper much more impactful. The authors should at least discuss this question in the Discussion.

We thank the reviewer for the suggestion. However also in the response to diluted urine the number of spikes was quite variable, as shown in fig 1B. Since the recording session is quite long we did not manage to perform in the same cell the current and the urine stimulation protocol, that would have allowed a more precise evaluation of the two adaptation mechanisms. We plan to further investigate this aspect.

We added this observation in the discussion, page 9 lines 319-323

Also, do the VSNs used for the current injection experiments pass the Fig 1 test?

We did not perform the test for chemical stimulation to the cells evaluated in current injection experiments. However the percentage of firing neurons that responded to diluted urine was quite high (>80%).

Second, in the experiments characterizing the slow inactivation of Na+ channels (Fig 7), Fig 7F curve should contain both the fast inactivation and the slow inactivation components. Why did the test pulse in Figure 7E set to 0 mV instead of -20 mV? If it were set at -20 mV, Fig 7F curve would be able to compare directly with Fig 7D curve, and the difference would indicate the slow inactivation. With the curve obtained at 0 mV, how could the authors know that the channel underwent slow inactivation?

The test potential should not affect the parameters of either slow or fast inactivation. To reduce variability we chose a potential at which the sodium current was maximally activated, and both -20 and 0 mV satisfy this criterion.

As reported at line 253, page 7, the repolarizing step at -80 mV before the step at 0 mV allows the complete recovery from fast inactivation allowing to distinguish fast and slow inactivation.

There are a few minor errors.

Line 155, “average input resistance was 1.3 {plus minus} 0.5 (n = 34)”. The resistance unit is missing.

We added the resistance unit

Line 185, “show-term” should be “short-term"

We corrected as suggested

Figure 7E, what does the “-100 mV” mean?

We corrected the Figure moving the “-100 mV” at the right position.

## References

[B1] Abbas F, Vinberg F (2021) Transduction and adaptation mechanisms in the cilium or microvilli of photoreceptors and olfactory receptors from insects to humans. Front Cell Neurosci 15:662453. 10.3389/fncel.2021.662453 33867944 PMC8046925

[B2] Amjad A, Hernandez-Clavijo A, Pifferi S, Maurya DK, Boccaccio A, Franzot J, Rock J, Menini A (2015) Conditional knockout of TMEM16A/anoctamin1 abolishes the calcium-activated chloride current in mouse vomeronasal sensory neurons. J Gen Physiol 145:285–301. 10.1085/jgp.201411348 25779870 PMC4380210

[B3] Bean BP (2007) The action potential in mammalian central neurons. Nat Rev Neurosci 8:451–465. 10.1038/nrn2148 17514198

[B4] Bolz F, Kasper S, Bufe B, Zufall F, Pyrski M (2017) Organization and plasticity of sodium channel expression in the mouse olfactory and vomeronasal epithelia. Front Neuroanat 11:28. 10.3389/fnana.2017.00028 28420967 PMC5376585

[B5] Brandão SC, Silies M, Martelli C (2021) Adaptive temporal processing of odor stimuli. Cell Tissue Res 383:125–141. 10.1007/s00441-020-03400-9 33404843 PMC7873106

[B6] Brennan PA (2010) Pheromones and mammalian behavior. In: The neurobiology of olfaction, Chap 6 (Menini A, ed), pp 157–179. Boca Raton, FL: CRC/Taylor and Francis. 21882427

[B7] De Palo G, Boccaccio A, Miri A, Menini A, Altafini C (2012) A dynamical feedback model for adaptation in the olfactory transduction pathway. Biophys J 102:2677–2686. 10.1016/j.bpj.2012.04.040 22735517 PMC3379019

[B8] Dibattista M, Amjad A, Maurya DK, Sagheddu C, Montani G, Tirindelli R, Menini A (2012) Calcium-activated chloride channels in the apical region of mouse vomeronasal sensory neurons. J Gen Physiol 140:3–15. 10.1085/jgp.201210780 22732308 PMC3382724

[B9] Doyle WI, Dinser JA, Cansler HL, Zhang X, Dinh DD, Browder NS, Riddington IM, Meeks JP (2016) Faecal bile acids are natural ligands of the mouse accessory olfactory system. Nat Commun 7:11936. 10.1038/ncomms11936 27324439 PMC4919516

[B10] Dulac C, Axel R (1995) A novel family of genes encoding putative pheromone receptors in mammals. Cell 83:195–206. 10.1016/0092-8674(95)90161-2 7585937

[B11] Eisinga R, Heskes T, Pelzer B, Te Grotenhuis M (2017) Exact p-values for pairwise comparison of Friedman rank sums, with application to comparing classifiers. BMC Bioinformatics 18:68. 10.1186/s12859-017-1486-2 28122501 PMC5267387

[B12] Fieni F, Ghiaroni V, Tirindelli R, Pietra P, Bigiani A (2003) Apical and basal neurones isolated from the mouse vomeronasal organ differ for voltage-dependent currents. J Physiol 552:425–436. 10.1113/jphysiol.2003.052035 14561826 PMC2343397

[B13] Fleidervish IA, Friedman A, Gutnick MJ (1996) Slow inactivation of Na+ current and slow cumulative spike adaptation in mouse and guinea-pig neocortical neurones in slices. J Physiol 493:83–97. 10.1113/jphysiol.1996.sp021366 8735696 PMC1158952

[B14] Francia S, Pifferi S, Menini A, Tirindelli R (2014) Vomeronasal receptors and signal transduction in the vomeronasal organ of mammals. In: Neurobiology of chemical communication, Chap 10 (Mucignat-Caretta C, ed), pp 297–329. Boca Raton, FL: CRC/Taylor and Francis. 24830038

[B15] Hernandez-Clavijo A, Sarno N, Gonzalez-Velandia KY, Degen R, Fleck D, Rock JR, Spehr M, Menini A, Pifferi S (2021) TMEM16A and TMEM16B modulate pheromone-evoked action potential firing in mouse vomeronasal sensory neurons. eNeuro 8:ENEURO.0179-21.2021. 10.1523/ENEURO.0179-21.2021 PMC8445037 34433575

[B16] Herrada G, Dulac C (1997) A novel family of putative pheromone receptors in mammals with a topographically organized and sexually dimorphic distribution. Cell 90:763–773. 10.1016/s0092-8674(00)80536-x 9288755

[B17] Holy TE, Dulac C, Meister M (2000) Responses of vomeronasal neurons to natural stimuli. Science 289:1569–1572. 10.1126/science.289.5484.1569 10968796

[B18] Ibarra-Soria X, Levitin MO, Saraiva LR, Logan DW (2014) The olfactory transcriptomes of mice. PLoS Genet 10:e1004593. 10.1371/journal.pgen.1004593 25187969 PMC4154679

[B19] Jenerick H (1963) Phase plane trajectories of the muscle spike potential. Biophys J 3:363–377. 10.1016/s0006-3495(63)86827-7 14062456 PMC1366455

[B20] Jung HY, Mickus T, Spruston N (1997) Prolonged sodium channel inactivation contributes to dendritic action potential attenuation in hippocampal pyramidal neurons. J Neurosci 17:6639–6646. 10.1523/JNEUROSCI.17-17-06639.1997 9254676 PMC6573150

[B21] Kaur AW, Ackels T, Kuo T-H, Cichy A, Dey S, Hays C, Kateri M, Logan DW, Marton TF, Spehr M, Stowers L (2014) Murine pheromone proteins constitute a context-dependent combinatorial code governing multiple social behaviors. Cell 157:676–688. 10.1016/j.cell.2014.02.025 24766811 PMC4051225

[B22] Kim KJ, Rieke F (2003) Slow Na+ inactivation and variance adaptation in salamander retinal ganglion cells. J Neurosci 23:1506–1516. 10.1523/JNEUROSCI.23-04-01506.2003 12598639 PMC6742238

[B23] Kim S, Ma L, Yu CR (2011) Requirement of calcium-activated chloride channels in the activation of mouse vomeronasal neurons. Nat Commun 2:365. 10.1038/ncomms1368 21694713 PMC3156823

[B24] Leinders-Zufall T, Storch U, Bleymehl K, Mederos Y Schnitzler M, Frank JA, Konrad DB, Trauner D, Gudermann T, Zufall F (2018) PhoDAGs enable optical control of diacylglycerol-sensitive transient receptor potential channels. Cell Chem Biol 25:215–223.e3. 10.1016/j.chembiol.2017.11.008 29276045

[B25] Liberles SD, Horowitz LF, Kuang D, Contos JJ, Wilson KL, Siltberg-Liberles J, Liberles DA, Buck LB (2009) Formyl peptide receptors are candidate chemosensory receptors in the vomeronasal organ. Proc Natl Acad Sci U|S|A 106:9842–9847. 10.1073/pnas.0904464106 19497865 PMC2690606

[B26] Liman ER, Buck LB (1994) A second subunit of the olfactory cyclic nucleotide-gated channel confers high sensitivity to cAMP. Neuron 13:611–621. 10.1016/0896-6273(94)90029-9 7522482

[B27] Liman ER, Corey DP (1996) Electrophysiological characterization of chemosensory neurons from the mouse vomeronasal organ. J Neurosci 16:4625–4637. 10.1523/JNEUROSCI.16-15-04625.1996 8764651 PMC6579035

[B28] Lucas P, Ukhanov K, Leinders-Zufall T, Zufall F (2003) A diacylglycerol-gated cation channel in vomeronasal neuron dendrites is impaired in TRPC2 mutant mice: mechanism of pheromone transduction. Neuron 40:551–561. 10.1016/s0896-6273(03)00675-5 14642279

[B29] Martelli C, Storace DA (2021) Stimulus driven functional transformations in the early olfactory system. Front Cell Neurosci 15:684742. 10.3389/fncel.2021.684742 34413724 PMC8369031

[B30] Matsunami H, Buck LB (1997) A multigene family encoding a diverse array of putative pheromone receptors in mammals. Cell 90:775–784. 10.1016/s0092-8674(00)80537-1 9288756

[B31] Mickus T, Jung HY, Spruston N (1999) Slow sodium channel inactivation in CA1 pyramidal cells. Ann N|Y Acad Sci 868:97–101. 10.1111/j.1749-6632.1999.tb11280.x 10414288

[B32] Mohrhardt J, Nagel M, Fleck D, Ben-Shaul Y, Spehr M (2018) Signal detection and coding in the accessory olfactory system. Chem Senses 43:667–695. 10.1093/chemse/bjy061 30256909 PMC6211456

[B33] Moss RL, Flynn RE, Shen XM, Dudley C, Shi J, Novotny M (1997) Urine-derived compound evokes membrane responses in mouse vomeronasal receptor neurons. J Neurophysiol 77:2856–2862. 10.1152/jn.1997.77.5.2856 9163402

[B34] Naundorf B, Wolf F, Volgushev M (2006) Unique features of action potential initiation in cortical neurons. Nature 440:1060–1063. 10.1038/nature04610 16625198

[B35] Nodari F, Hsu F-F, Fu X, Holekamp TF, Kao L-F, Turk J, Holy TE (2008) Sulfated steroids as natural ligands of mouse pheromone-sensing neurons. J Neurosci 28:6407–6418. 10.1523/JNEUROSCI.1425-08.2008 18562612 PMC2726112

[B36] Rupasinghe DB, Knapp O, Blomster LV, Schmid AB, Adams DJ, King GF, Ruitenberg MJ (2012) Localization of Nav 1.7 in the normal and injured rodent olfactory system indicates a critical role in olfaction, pheromone sensing and immune function. Channels (Austin) 6:103–110. 10.4161/chan.19484 22622154

[B37] Ryba NJ, Tirindelli R (1997) A new multigene family of putative pheromone receptors. Neuron 19:371–379. 10.1016/S0896-6273(00)80946-0 9292726

[B38] Shimazaki R, Boccaccio A, Mazzatenta A, Pinato G, Migliore M, Menini A (2006) Electrophysiological properties and modeling of murine vomeronasal sensory neurons in acute slice preparations. Chem Senses 31:425–435. 10.1093/chemse/bjj047 16547196

[B39] Silva J (2014) Slow inactivation of Na(+) channels. Handb Exp Pharmacol 221:33–49. 10.1007/978-3-642-41588-3_3 24737231

[B40] Silvotti L, Cavaliere RM, Belletti S, Tirindelli R (2018) In-vivo activation of vomeronasal neurons shows adaptive responses to pheromonal stimuli. Sci Rep 8:8490. 10.1038/s41598-018-26831-5 29855521 PMC5981476

[B41] Spehr J, Hagendorf S, Weiss J, Spehr M, Leinders-Zufall T, Zufall F (2009) Ca^2+^-calmodulin feedback mediates sensory adaptation and inhibits pheromone-sensitive ion channels in the vomeronasal organ. J Neurosci 29:2125–2135. 10.1523/JNEUROSCI.5416-08.2009 19228965 PMC6666346

[B42] Stowers L, Holy TE, Meister M, Dulac C, Koentges G (2002) Loss of sex discrimination and male-male aggression in mice deficient for TRP2. Science 295:1493–1500. 10.1126/science.1069259 11823606

[B43] Tirindelli R (2021) Coding of pheromones by vomeronasal receptors. Cell Tissue Res 383:367–386. 10.1007/s00441-020-03376-6 33433690

[B44] Tirindelli R, Dibattista M, Pifferi S, Menini A (2009) From pheromones to behavior. Physiol Rev 89:921–956. 10.1152/physrev.00037.2008 19584317

[B45] Toib A, Lyakhov V, Marom S (1998) Interaction between duration of activity and time course of recovery from slow inactivation in mammalian brain Na^+^ channels. J Neurosci 18:1893–1903. 10.1523/JNEUROSCI.18-05-01893.1998 9465014 PMC6792612

[B46] Torre V, Ashmore JF, Lamb TD, Menini A (1995) Transduction and adaptation in sensory receptor cells. J Neurosci 15:7757–7768. 10.1523/JNEUROSCI.15-12-07757.1995 8613717 PMC6577959

[B47] Touhara K, Vosshall LB (2009) Sensing odorants and pheromones with chemosensory receptors. Annu Rev Physiol 71:307–332. 10.1146/annurev.physiol.010908.163209 19575682

[B48] Ukhanov K, Leinders-Zufall T, Zufall F (2007) Patch-clamp analysis of gene-targeted vomeronasal neurons expressing a defined V1r or V2r receptor: ionic mechanisms underlying persistent firing. J Neurophysiol 98:2357–2369. 10.1152/jn.00642.2007 17715188

[B49] Wark B, Lundstrom BN, Fairhall A (2007) Sensory adaptation. Curr Opin Neurobiol 17:423–429. 10.1016/j.conb.2007.07.001 17714934 PMC2084204

[B50] Webster MA (2012) Evolving concepts of sensory adaptation. F1000 Biol Rep 4:21. 10.3410/B4-21 23189092 PMC3501690

[B51] Wong WM, Nagel M, Hernandez-Clavijo A, Pifferi S, Menini A, Spehr M, Meeks JP (2018) Sensory adaptation to chemical cues by vomeronasal sensory neurons. eNeuro 5:ENEURO.0223-18.2018. 10.1523/ENEURO.0223-18.2018 PMC6088365 30105301

[B52] Wysocki CJ, Nyby J, Whitney G, Beauchamp GK, Katz Y (1982) The vomeronasal organ: primary role in mouse chemosensory gender recognition. Physiol Behav 29:315–327. 10.1016/0031-9384(82)90021-x 7146137

